# Feasibility of an endoscope-dominated side-to-end hypoglossal-facial anastomosis: an anatomical study

**DOI:** 10.3389/fsurg.2023.1251527

**Published:** 2023-08-21

**Authors:** Xiaobing Yang, Dulegeqi Man, Yang Yang, Xingang Li

**Affiliations:** ^1^ Department of Neurosurgery, Qilu Hospital, Cheeloo College of Medicine and Institute of Brain and Brain-Inspired Science, Shandong University, Jinan, China; ^2^ Jinan Microecological Biomedicine Shandong Laboratory and Shandong Key Laboratory of Brain Function Remodeling, Jinan, China; ^3^ Department of Neurosurgery, International Mongolia Hospital of Inner Mongolia, Hohhot, China

**Keywords:** hypoglossal-facial anastomosis, transaditus approach, endoscopic, mastoidectomy, facial nerve reanimation

## Abstract

**Objective:**

A surgical simulation of an endoscope-dominated side-to-end hypoglossal-facial anastomosis was performed to evaluate the feasibility.

**Methods:**

Eight anatomical cadaver heads (16 sides) were recruited. The steps in conventional procedures were abbreviated or omitted. A facial nerve was first harvested near its external genu and was used for a side-to-end hypoglossal-facial anastomosis. The stump of the used facial nerve was truncated and recycled immediately caudal to the facial recess in another anastomosis and then recycled again at the stylomastoid foramen. As a recycled stump becomes too short to ensure a side-to-end anastomosis, the hypoglossal nerve was transected *in situ*, and an endoscopic end-to-end hypoglossal-facial anastomosis was attempted. Surgical simulation and quantitative measurement methods were used to analyze the anastomosis effects of different harvested sites of the facial nerve.

**Results:**

Several steps in the conventional procedures provide little benefit in endoscopic surgery. A facial nerve stump recycled at the stylomastoid foramen is too short to ensure a tensionless side-to-end anastomosis. An endoscopic end-to-end hypoglossal-facial anastomosis was feasible, although it required more time than the classical microsurgical anastomosis. The greater agility of an endoscope enables the conventional surgical steps to be overlapped or interweaved into the procedure.

**Conclusions:**

The multiple surgical fields and ability to manipulate the viewpoint provided by an endoscope have brought about breakthroughs in classical surgical paradigms. In addition, it is best to choose the sites of the facial nerve harvested near the external genu. If unavailable, an alternative section site could be selected immediately caudal to the facial recess, but cannot be distal to the stylomastoid foramen. The length of the stump should be individualized and preferably optimized with a nerve stimulator.

## Introduction

Facial paralysis is a disabling clinical syndrome (1). Despite the major advances in microscopic surgery (2, 3), facial nerve repair remains a significant challenge for neurosurgeons.

An end-to-end facial nerve repair allows the best nerve function recovery in patients with facial nerve transection, in cases where the proximal and distal nerve ends are close (4, 5). When the proximal facial nerve stump is not available for an *in situ* anastomosis (5–9), such as in the case of a facial nerve rupture near the cerebellopontine angle (CPA) area, an end-to-end hypoglossal-facial nerve anastomosis has been used to recover the facial nerve function. However, this surgery is often associated with significant speech disorder and tongue atrophy as side effects (5). To minimize these side effects, Darrouzet et al. (7, 9–11) published a new microscopic side-to-end hypoglossal-facial neurorrhaphy technique in 1997.

Endoscope-facilitated surgery has been widely used to improve the surgical outcomes, with the advantages of broad surgical fields, multiple surgical views, and the ability to quickly switch between different surgical fields and surgical targets. To study the feasibility and potential advantages of an endoscope-dominated side-to-end hypoglossal-facial nerve anastomosis technique, we performed a surgical simulation study using eight human cadaver specimens (16 sides).

## Materials and methods

Eight cadaver heads (16 sides) were used for the surgical simulation of an endoscope-dominated side-to-end hypoglossal-facial anastomosis technique. Statistical analysis was performed using a paired Student’s *t*-test for two-group comparison using GraphPad Prism 9.5 (La Jolla, CA, USA). The *P*-values of <0.05 were considered to be statistically significant.

## Surgical techniques and results

**Figure 1:**
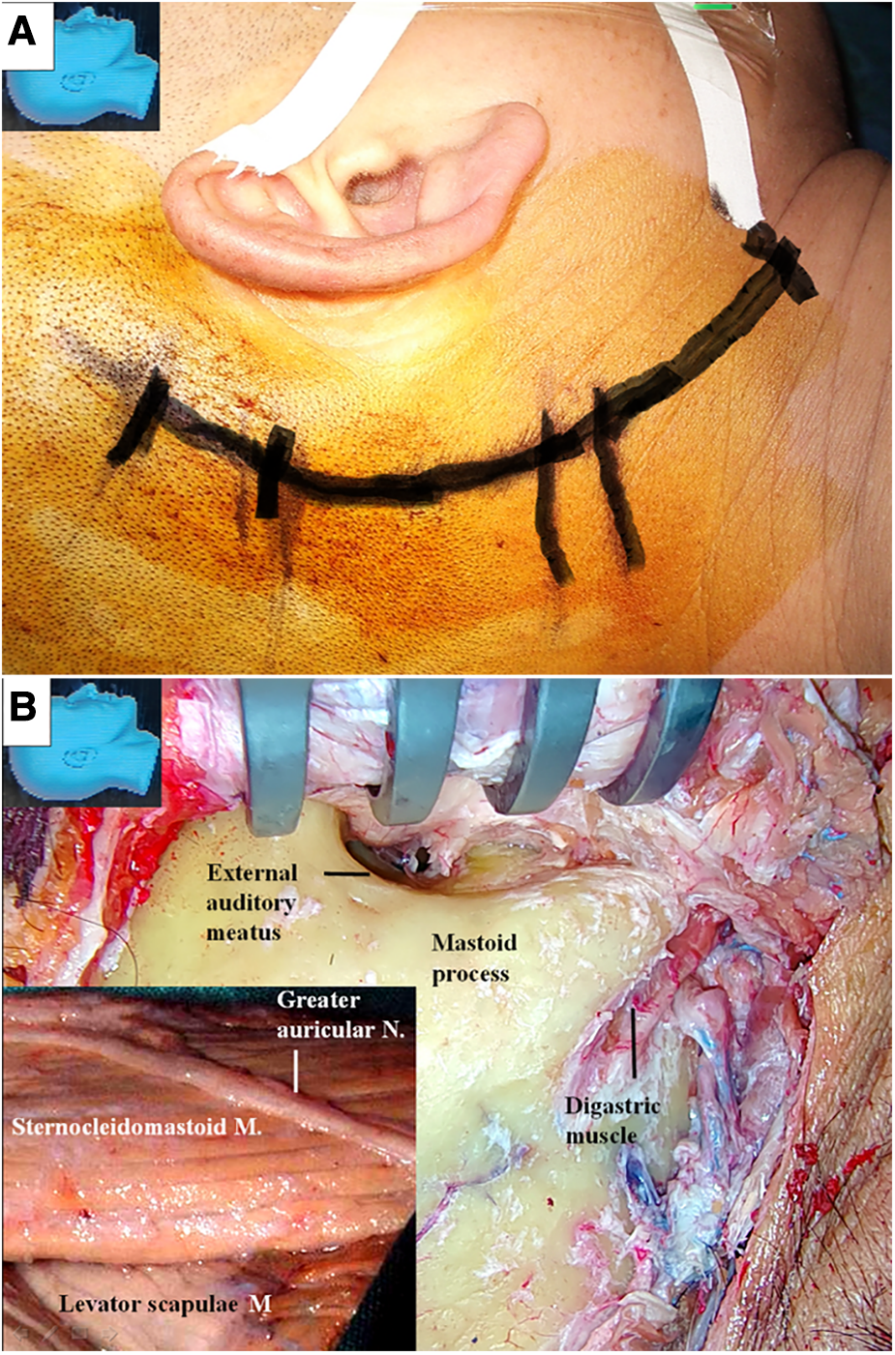
Incision and the exposure of the mastoid process. (**A**) A retroauricular-arch incision is made, exposing the mastoid process, extending it caudally along the anterior border of the sternocleidomastoid muscle until just above the angle of the mandible; (**B**) the greater auricular nerve is dissected and preserved (inlet). The sternocleidomastoid muscle is elevated from the mastoid process and posteriorly retracted. The mastoid tip is exposed by detaching the muscle from its surface. M, muscle; N, nerve.

A retroauricular-arch incision was made, exposing the mastoid process and extending caudally along the anterior border of the sternocleidomastoid muscle until just above the angle of the mandible (Figure 1A) (12). Attention was given to avoid injury to the greater auricular nerve (Figure 1B). The sternocleidomastoid muscle was detached from the mastoid process and posteriorly retracted to expose the mastoid tip (Figure 1B).

From here on, the surgical simulations were predominantly performed with endoscopic techniques.

**Figure 2:**
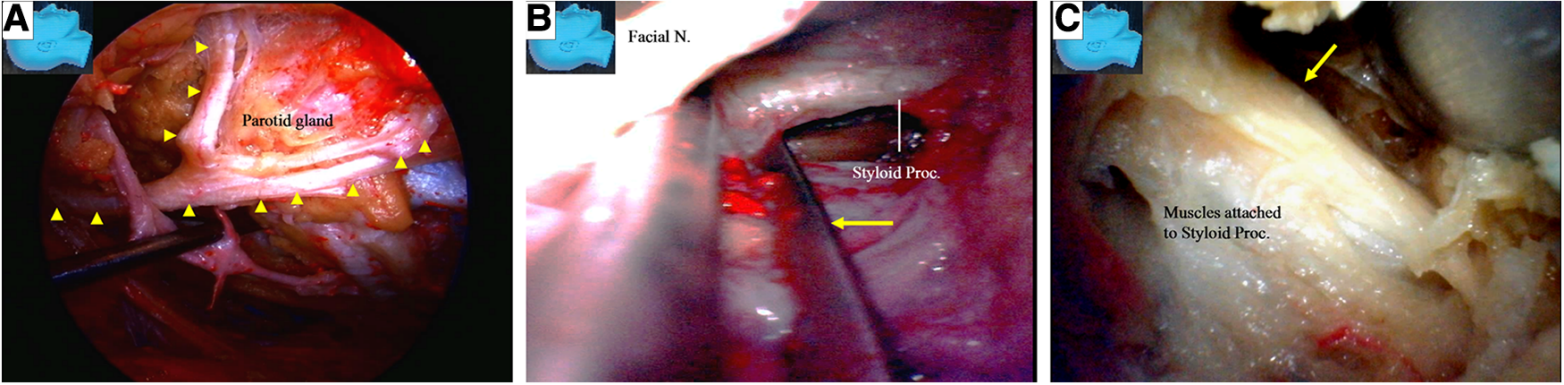
Endoscopic view: the facial nerve location, identification, and management at the cranial base. (**A**) The facial nerve and its branches (arrowheads) are located and identified. (**B**) Styloid process is being located and held within a forceps; (**C**) fracturing the styloid process (arrow) and dealing with the attached muscle may be performed much more easily with an endoscope than with an operative microscope. But the necessity and benefit of this step was challenged in the practice of endoscopic dissection. N, nerve; Proc, process.

Using an endoscope, the facial nerve was exposed in the cranial base and within the parotid gland. As illustrated in Figure 2, we found that surgical exposure of the facial nerve trunk and its distal portion within the parotid gland (Figure 2A) could be achieved more easily and less traumatically with the aid of an endoscope.

In microscopic surgery, the styloid process (Figures 2B,C) is traditionally used as a landmark for localizing the stylomastoid foramen and the main trunk of the facial nerve (12, 13). Using an endoscope, we found that the facial nerve and its branches were located more superficially than the styloid process (Figures 2B,C), and we could easily identify them without using the styloid process as a landmark, both in the stylomastoid foramen area (Figure 2C) and within the parotid gland (Figure 2E). With the endoscope, we found that the stylomastoid foramen could be easily identified by tracing along the facial nerve and its branches (Figure 2F). We kept the styloid process intact during the endoscopic surgery. We found that the identification, fracture (Figures 2B,C), and retraction of the styloid process with attached muscles, which is required during microscopic surgery, could also be performed easily with the endoscope technique, if needed.

**Figure 3:**
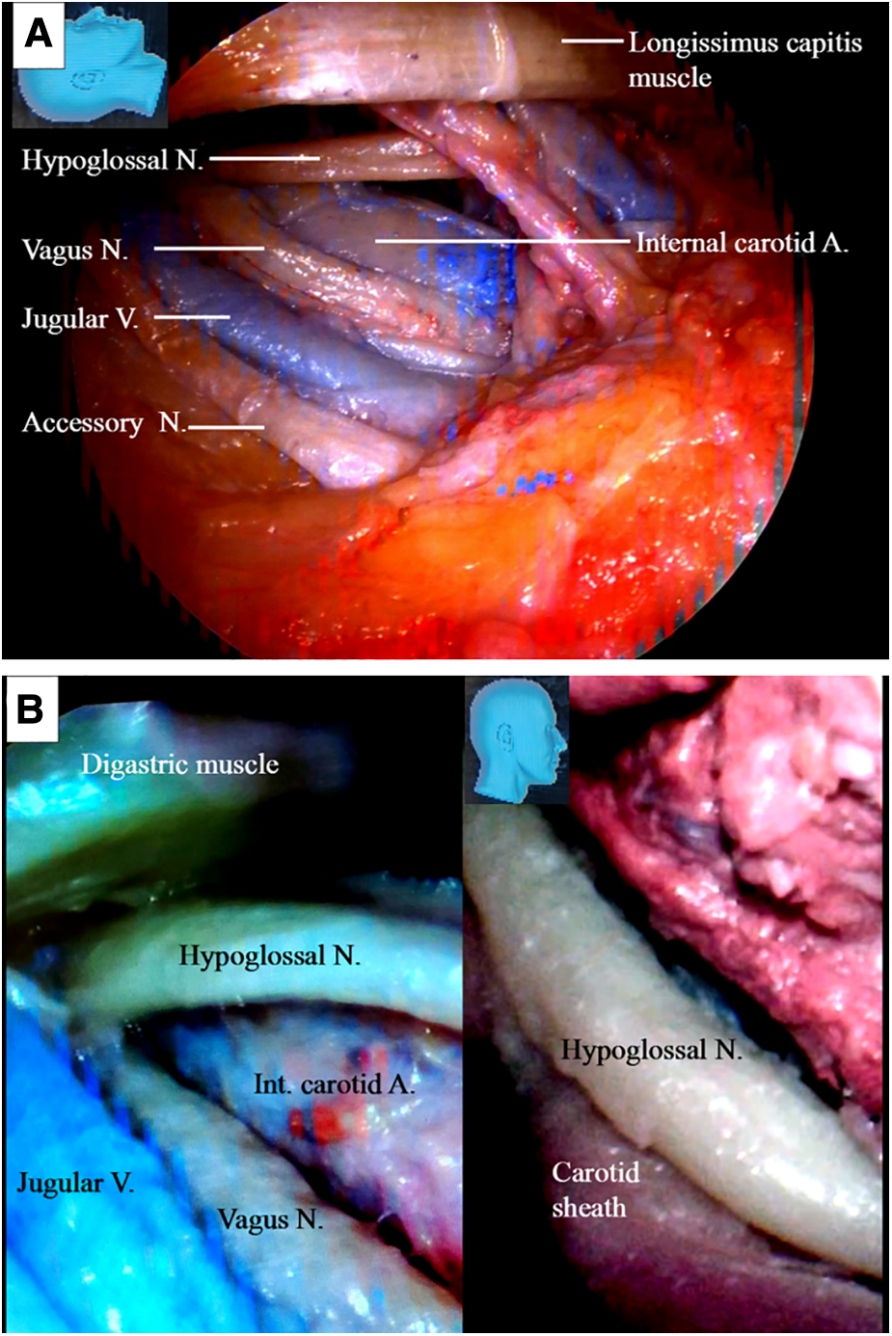
Endoscopic view of the hypoglossal nerve and its surrounding structures. (**A**) The surrounding key structures having intimated relationship with the hypoglossal nerve in the cervical region; (**B**) left: a view underneath the digastric muscle. The hypoglossal nerve is found deep in the posterior belly of the digastric muscle at the caudal end of the incision. Injury to the crucial structures around the hypoglossal nerve should be avoided; right: the carotid sheath should be kept intact as the hypoglossal nerve is managed. A, artery; V, vein; N, nerve.

Under endoscopy, the hypoglossal nerve and its neighboring structures were exposed, as illustrated in (Figure 3). The nerve was located deeper than the posterior belly of the digastric muscle near the caudal end of our surgical incision. During the endoscopic surgery, the carotid sheath remained intact (Figure 3B).

**Figure 4:**
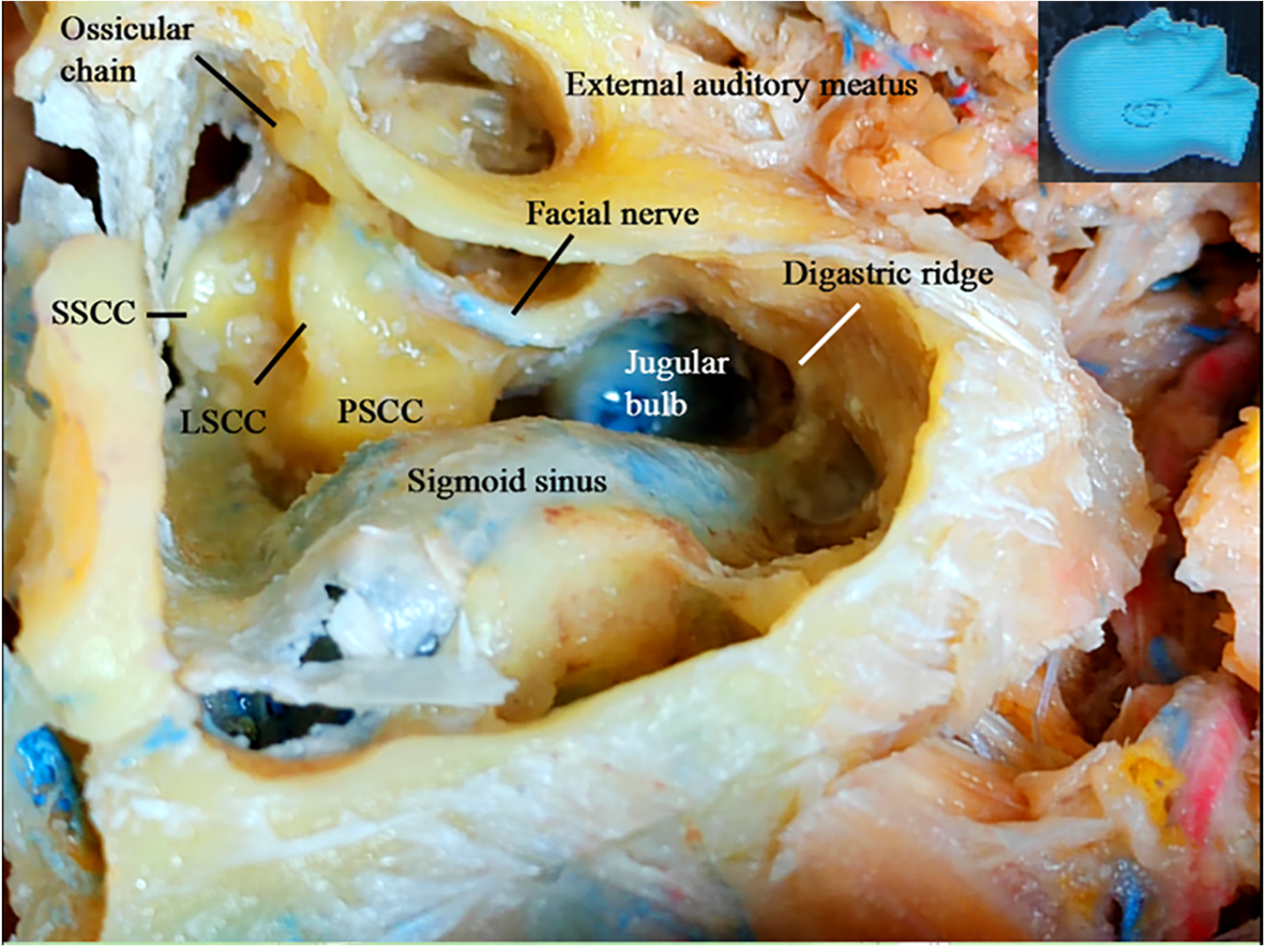
Gross anatomy: the structures exposed following a classical mastoidectomy. LSCC, lateral semicircular canal; SSCC, superior semicircular canal; PSCC, posterior semicircular canal.

The structures exposed in classic mastoidectomy are shown in Figure 4 (12, 13). We performed a simulated endoscope-dominated transaditus hypoglossal-facial nerve anastomosis surgery with some modifications, exploiting the advantages of the endoscope, including the broad surgical fields, multiple surgical views, and ability to quickly switch among different surgical fields.

**Figure 5:**
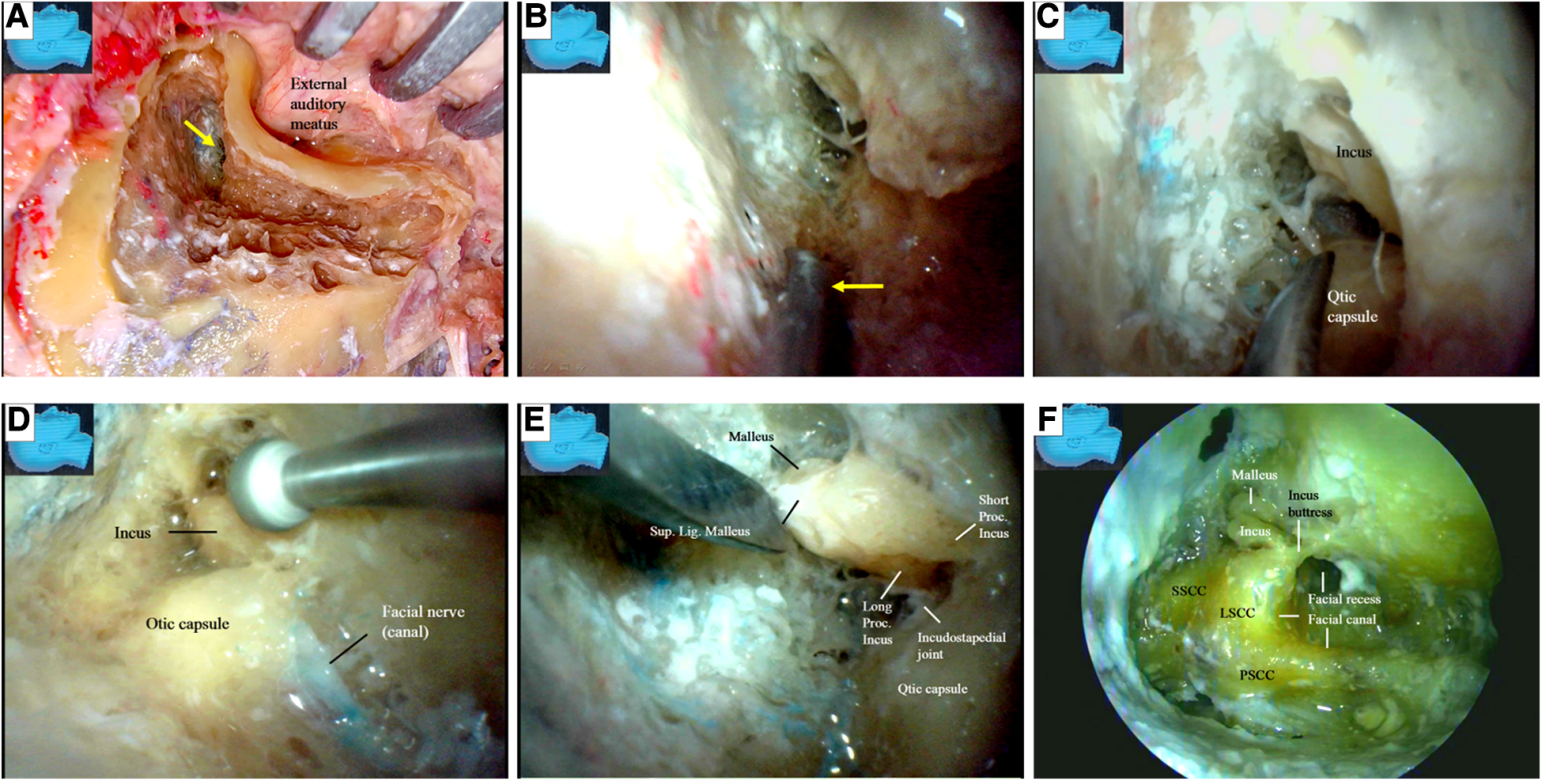
Endoscopic view: modified transaditus approach. (**A**) Partial cortical mastoidectomy is performed, and an endoscope was introduced once the antrum (arrow) is encountered; (**B**) followed by an endoscope, a dissector (arrow) is advancing along the aditus ad antrum, a large irregular opening connecting the antrum and the tympanic cavity; (**C**) the ossicular chain (incus) and the otic capsule (semicircular canals) have come into view, which are inaccessible for a surgical microscope; (**D**) the ossicular chain (incus) and the otic capsule (semicircular canals) have been verified, and the surgical corridor is being widened with a diamond drill to accommodate both the endoscope and the surgical instruments; (**E**) as the ossicular chain is approached, the surrounding structures have become more clear; (**F**) the semicircular canals are discernable in this specimen as observed in a conventional microsurgical dissection. We never skeletonize a superior or posterior semicircular canal when performing an endoscopic surgical simulation. LSCC, lateral semicircular canal; SSCC, superior semicircular canal; PSCC, posterior semicircular canal; Lig, ligament; Proc, process; Sup, superior.

We first performed a partial cortical mastoidectomy with a diamond drill. After the tympanic (mastoid) antrum was reached (Figure 5A), the endoscope was advanced along the aditus ad antrum (a large irregular opening connecting the antrum and the tympanic cavity) (Figure 5B) (14) to search for the ossicular chain, especially the incus, a key landmark (15) for the facial nerve (14–16). After the ossicular chain came into view (Figure 5C), the ossicular chain (incus) and the otic capsule (semicircular canals) were identified, and the surgical corridor was widened with a diamond drill to better accommodate the endoscope and other surgical instruments (Figure 5D).

**Figure 6:**
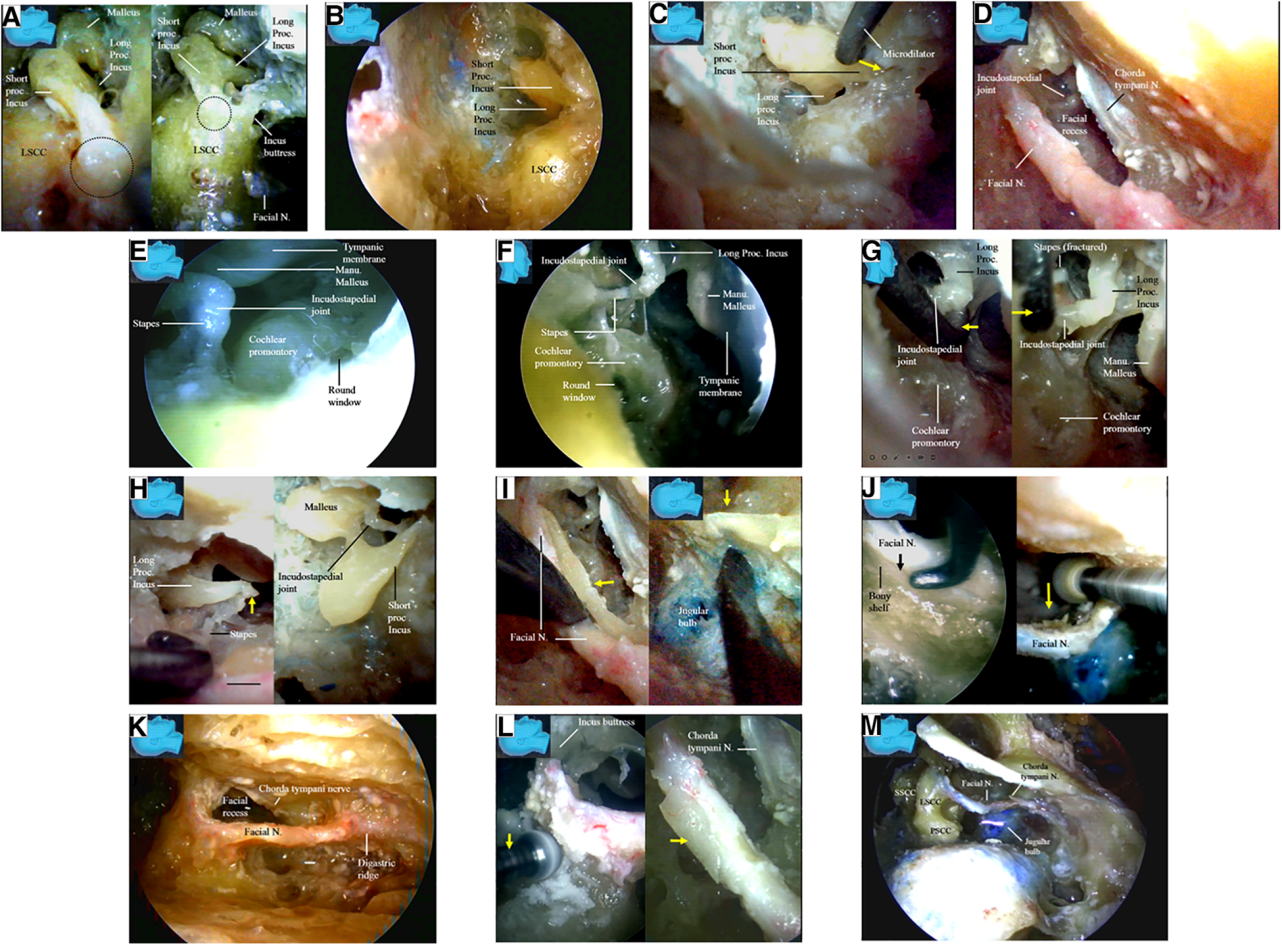
Endoscopic view: endoscopic inspection of the structures behind the facial nerve. (**A**) The fossa incudis (dash circle), a small depression in which the short process of the incus fits (left), has an intimated relationship with the facial canal (right); (**B**) the fossa incudis was inspected from above the facial recess; (**C**) a microdilator is advancing along the short process of the incus toward the fossa incudis (arrow); (**D**) the facial recess was a valuable access to the structures hidden behind the facial nerve; (**E**) an endoscope is entering and inspecting the facial recess; (**F**) a detailed observation of the structures directly underneath the facial nerve is made; (**G**) a microdilator (arrow) is fracturing the incudostapedial joint by force (left) to result in dislocation of the joint (right) via facial recess; (**H**) the higher mobility and agility of an endoscope enables the frequent and rapid reciprocal observation and manipulation among the different surgical fields. The incudostapedial joint is dislocated, broken, and torn via the facial recess approach (left) whereas the incus is removed from above via the fossa incudis (right), respectively; (**I**) a bony shelf (arrow) supporting the facial nerve from beneath the bottom of the canal (left) may be observed with the endoscope in all the specimens in this series, even in those with highly pneumatized labyrinthine, which make the jugular bulb completely exposed without a layer of bony shell (right); (**J**) anatomical relationship between the facial nerve and the jugular bulb: an interface (arrow) between the facial nerve and a bony shelf at the bottom of the canal is retrievable with the endoscope in all the specimens in this series (left). An endoscope intimately following the diamond drill enables the whole procedure under direct vision, and even the deep aspect of the facial canal is within the vision (right); (**K**) the facial nerve is skeletonized from the second (external) genu to the stylomastoid foramen; (**L**) the facial canal is skeletonized with a diamond drill (arrow) (left). Only a thin layer of bone (arrow) was left over the skeletonized facial nerve (right). The soft tissues, including the vessels in the facial canal and the nerve sheath, are left intact to preserve vascular supply to the facial nerve. The chorda tympani nerve have to be sectioned (arrows) to mobilize the facial nerve; (**M**) the chorda tympani has been sectioned, and the facial nerve is mobilized. Lig, ligament; LSCC, lateral semicircular; Manu, manubrium; N, nerve; Proc, process; Sup, superior canal; SSCC, superior semicircular canal; PSCC, posterior semicircular canal.

After the ossicular chain was located, the relationship of several key structures became clear (Figure 5E). The lateral semicircular canal and the incus buttress (Figure 5F) (16) were exposed to localize the facial nerve (canal). The otic capsule, or all three semicircular canals and the facial nerve (canal), were then identified in a highly pneumatized labyrinth, similar to the view in a classical microsurgical dissection (Figure 5F). We did not skeletonize the superior or posterior semicircular canal. We found that many important structures not well visualized with an operating microscope could be well visualized using an endoscope. The fossa incudis (a small depression bordered by the facial recess, in which the short process of the incus fits) (Figure 6A) was visualized above the facial recess (Figures 6B,C). Bordered superiorly by the fossa incudis, medially by the facial nerve, and laterally by the chorda tympani (15), the facial recess (Figure 6D) provided valuable access to visualize the structures hidden behind the facial nerve, including the incus, stapes (Figures 6E–H), malleus, tympanic membrane, and round window (Figures 6E,F).

The transaditus approach (14) with dislocation of the incudostapedial joint (Figures 6G,H) and removal of the incus (Figure 6H) was endoscopically simulated (Figures 6G,H). Using the endoscope’s ability to quickly switch between surgical fields, the incudostapedial joint was dislocated, broken, and torn (Figures 6G,H) via a facial recess approach, and the incus was removed from above using a fossa incudis approach (Figure 6H). However, we found that these surgical steps were not necessary for harvesting the facial nerve stumps (Figures 6D–H) under endoscopy. In fact, we recommend leaving the incus intact and using its short process as a landmark to localize the facial recess and the nerve (canal) (15). In addition, we kept the posterior incudal ligament (Figure 6A) [theoretically (16) serving as a soft tissue pillow to protect the ossicular chain from the vibrating effect of the rotating burr] and the incus buttress (Figure 6A) (16) (Figure 8B) intact during our endoscopic simulation.

The jugular bulb was located immediately medial to the facial nerve canal, with two-thirds posterior to and one-third anterior to the facial nerve (canal). Great care was taken to avoid injury to this important structure. A bony shelf supporting the facial nerve from beneath the bottom of the canal was observed under endoscopy in all specimens, even in those with a highly pneumatized labyrinth (Figure 6I). A clear interface (arrow) was found between the facial nerve and the bony shelf at the bottom of the canal. Under endoscopy, the whole surgical field could be viewed directly, including the deep aspect of the facial canal (Figure 6J), which was very helpful for minimizing potential inadvertent injury to the jugular bulb.

Under endoscopy, the facial nerve was skeletonized from the second (external) genu (12, 13) to the stylomastoid foramen (Figure 6K), leaving behind only a thin layer of bone over the facial nerve, which was removed later with a microdissector (Figure 6L). The soft tissues, including the vessels in the facial canal and the nerve sheath, were left intact to preserve the vascular supply to the facial nerve (Figure 6L).

**Figure 7:**
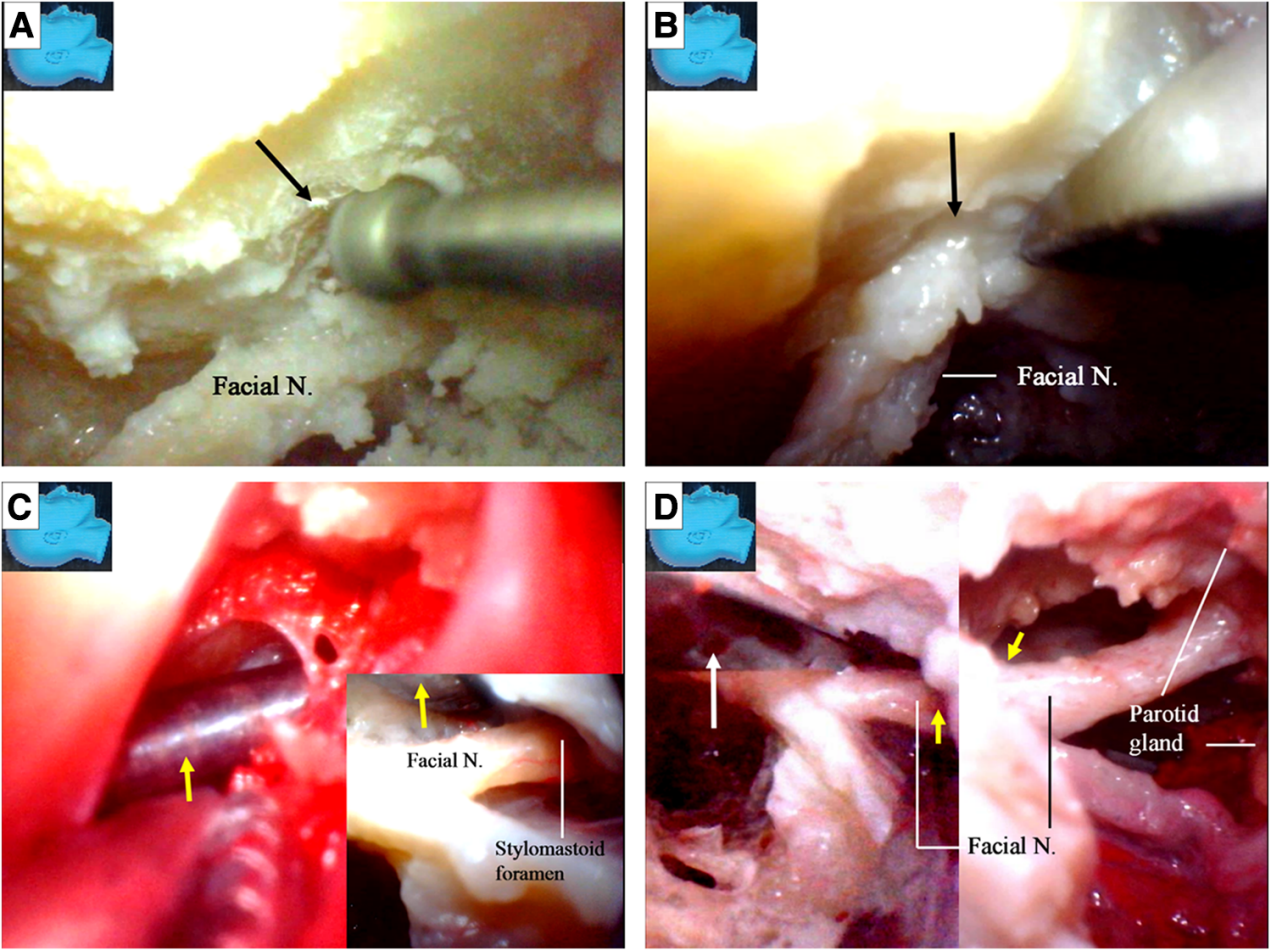
Endoscopic view: facial nerve transposition. (**A**) The stylomastoid foramen was opened (arrow) and widened with the diamond drill; (**B**) the facial nerve is released from the surrounding connective tissues within the opened stylomastoid foramen (arrow); (**C**) with the similar rapid reciprocation strategy between various surgical fields, a dissector (arrow) is inserted from within (inlet) and passed out of the opened stylomastoid foramen as a guidance from outside; (**D**) movements of slight manipulations from a forceps (white arrow) at the facial nerve inside the stylomastoid foramen (left) are transmitted out along the nerve and observed outside the foramen (yellow arrow) simultaneously (right). N, nerve.

**Figure 8:**
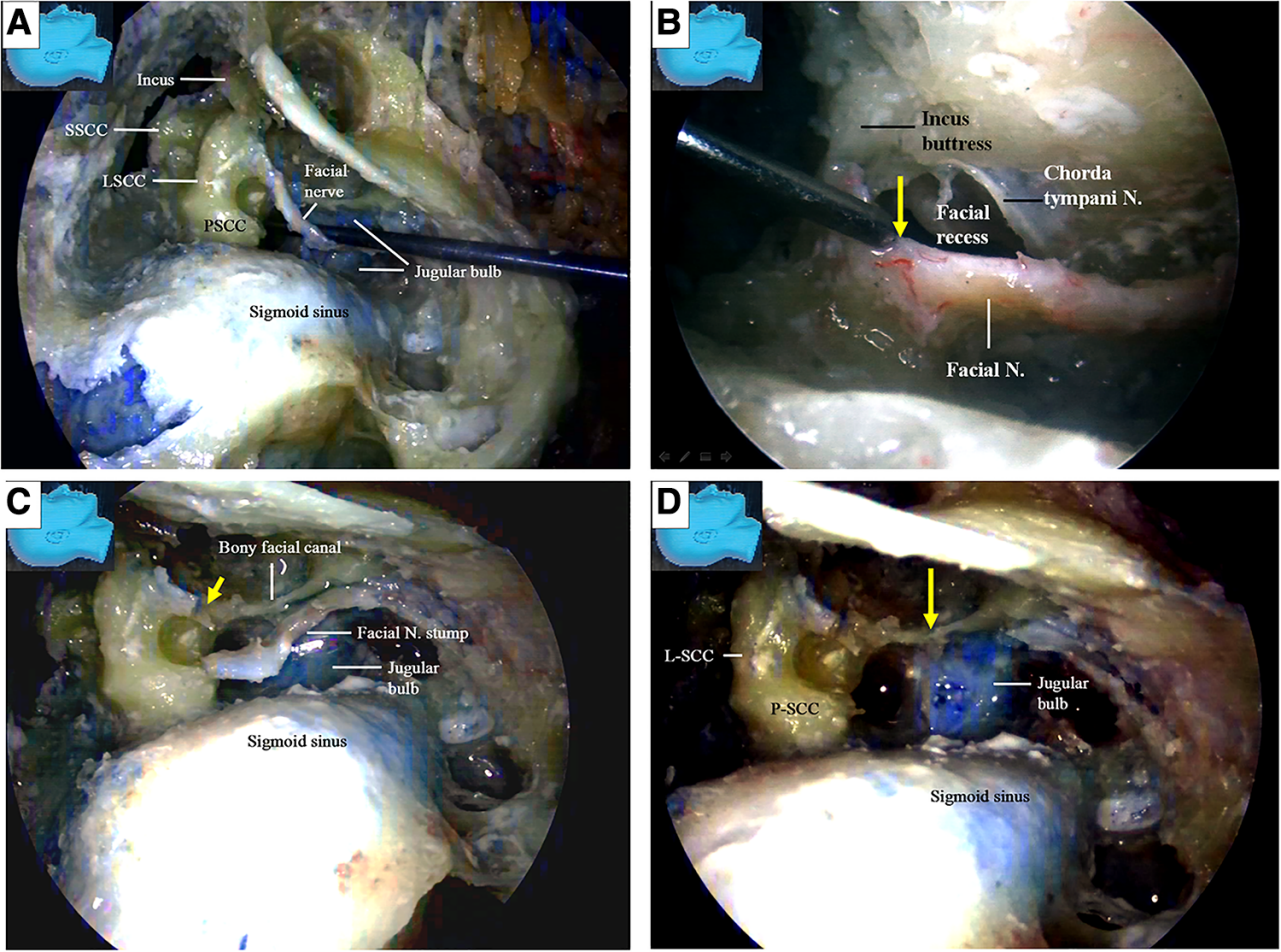
Endoscopic view: facial nerve transposition and harvest. (**A**) After obtaining a length of the facial nerve sufficient to perform a tensionless anastomosis, the most proximal portion is to be transected and harvested; (**B**) the most proximal portion is to be sectioned near its external genu (arrow) (left), several millimeters distal to the incus buttress (arrow) (right); (**C**) the facial nerve has been sectioned, and the distal portion of the nerve stump is harvested, peeled out of its bony canal, and displaced caudally; (**D**) the remnants (a bony shelf beneath the bottom of the canal) of the bony facial canal (arrow) is left *in situ* after the nerve stump has been harvested. LSCC, lateral semicircular canal; SSCC, superior semicircular canal; PSCC, posterior semicircular canal; N, nerve.

**Figure 9:**
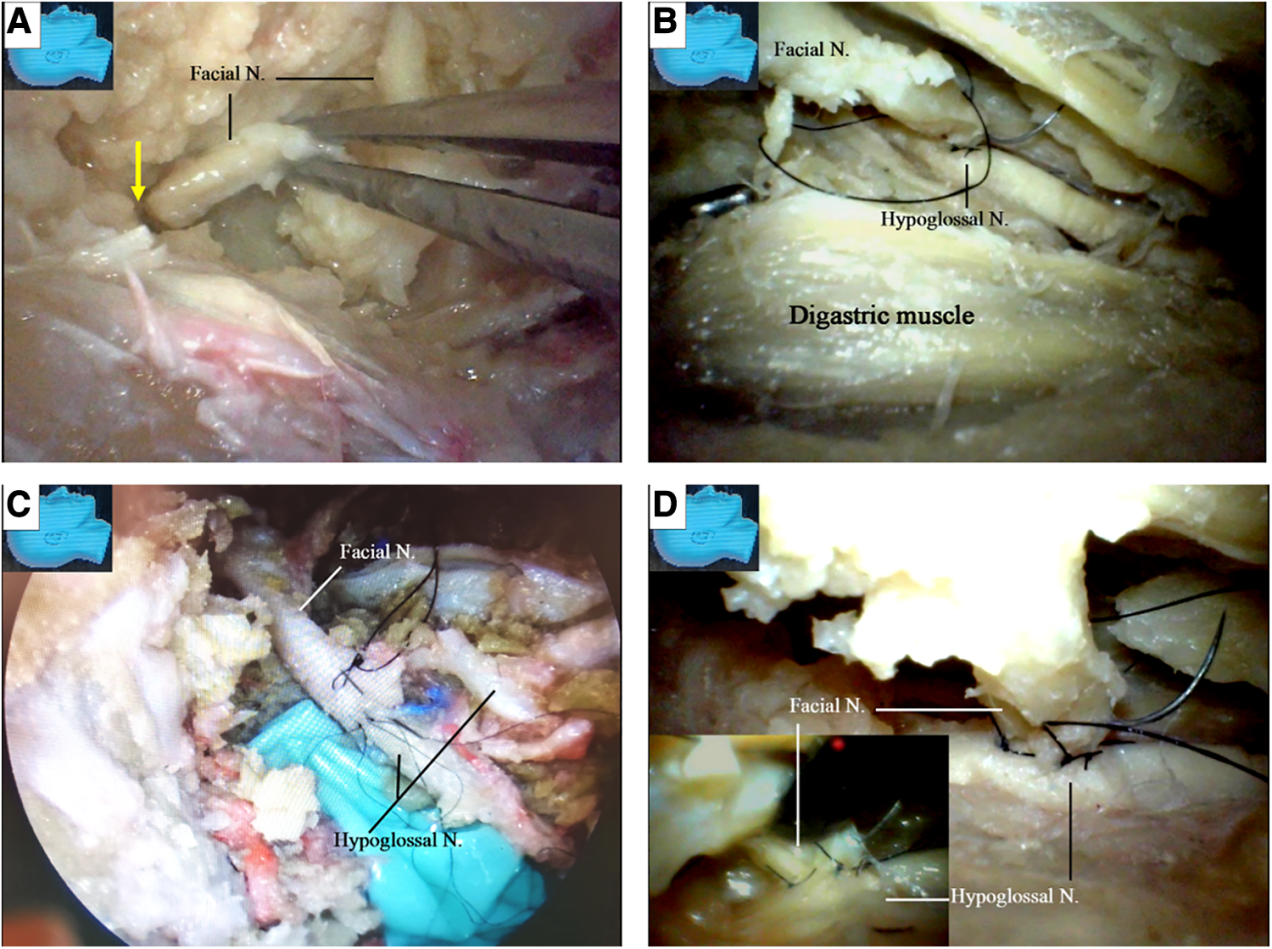
Endoscopic view: the side-to-end hypoglossal-facial anastomosis. (**A**) The stump of the facial nerve is taken out of the stylomastoid foramen (arrow); (**B**) the facial nerve is passed beneath the digastric muscle without tension, and a side-to-end hypoglossal-facial anastomosis with 11/0 nylon sutures is performed under a surgical microscope, scrutinized with an endoscope from various viewpoints; (**C**) an endoscopic view of the anastomosis performed under an operating microscope with 11/0 nylon sutures; (**D**) the endoscope is advancing underneath the facial nerve, an angle inaccessible for an operating microscope (inlet). N, nerve.

The chorda tympani nerve (Figures 6L,M) was sectioned to mobilize the facial nerve, and the stylomastoid foramen was opened and widened with a diamond drill (Figure 7A) to release the facial nerve from the surrounding tissues within the foramen (Figure 7B). Under endoscopy, a dissector was inserted into and then passed through and out of the opened stylomastoid foramen, using it as a mark of the foramen (Figure 7C). Forceps were used to gently move the facial nerve inside the stylomastoid foramen to help identify the distal part of the facial nerve outside the stylomastoid foramen (Figure 7D) by recognizing its movement (clinically, this can be accomplished by using a nerve stimulator). When a sufficient length of the facial nerve for a tensionless anastomosis was identified (Figure 8A), the nerve was transected at its most proximal portion near the external genu (12, 13) and at a site several millimeters distal to the incus buttress (Figure 8B). The distal portion of the nerve stump was harvested, peeled off the bony canal, and displaced caudally (Figure 8C) toward the previously exposed hypoglossal nerve (Figure 3). The remnants of the bony facial canal (the bony shelf beneath the bottom of the canal) were left *in situ* (Figures 8D,E). The facial nerve stump was then taken out of the stylomastoid foramen (Figures 9A,B) and trimmed for anastomosis. A longitudinal neurotomy was made along the lateral wall of the hypoglossal nerve, and the trimmed facial nerve stump was passed beneath the digastric muscle without tension (Figure 9C). A side-to-end hypoglossal-facial anastomosis was performed with 11/0 nylon sutures under an operating microscope, facilitated by the multiple-angled surgical views provided by the endoscope (Figures 9B–D). We found that the endoscope could provide access to many areas and many surgical views that could not be obtained with an operating microscope alone, such as the view from beneath the facial nerve (Figure 9D).

**Figure 10:**
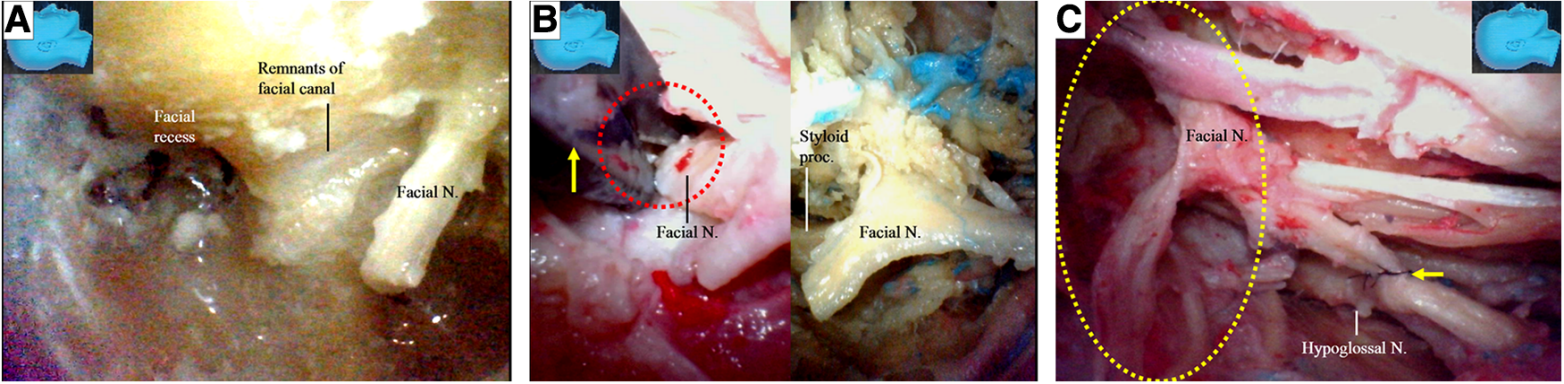
Endoscopic view. The facial nerve section at other sites for the facial nerve stump harvest; (**A**) a previously used facial nerve stump is truncated and harvested again immediately caudal to the facial recess; (**B**) the facial nerve is sectioned with a micro-scissors (arrow) at the stylomastoid foramen (dash circle) (left). The stump was then harvested, trimmed, and prepared for anastomosis (right); (**C**) the side-to-end hypoglossal-facial anastomosis is completed with 8/0 instead of 11/0 nylon sutures as relatively high tension between the nerves even exaggerating dissection (dash circle) had been performed. N, nerve, Proc, process.

The facial nerve stumps from other cutting sites (12, 13) were used for further side-to-end hypoglossal-facial anastomosis simulations. The used facial nerve stumps were placed back in their original positions, cut immediately caudal to the facial recess area, and used as new nerve stumps for further side-to-end hypoglossal-facial anastomosis simulations (Figure 10A). If the stumps still had sufficient length for a new anastomosis, they were used again and cut near the stylomastoid foramen (Figures 7D, 10B). More similar anastomosis simulations were performed.

**Figure 11:**
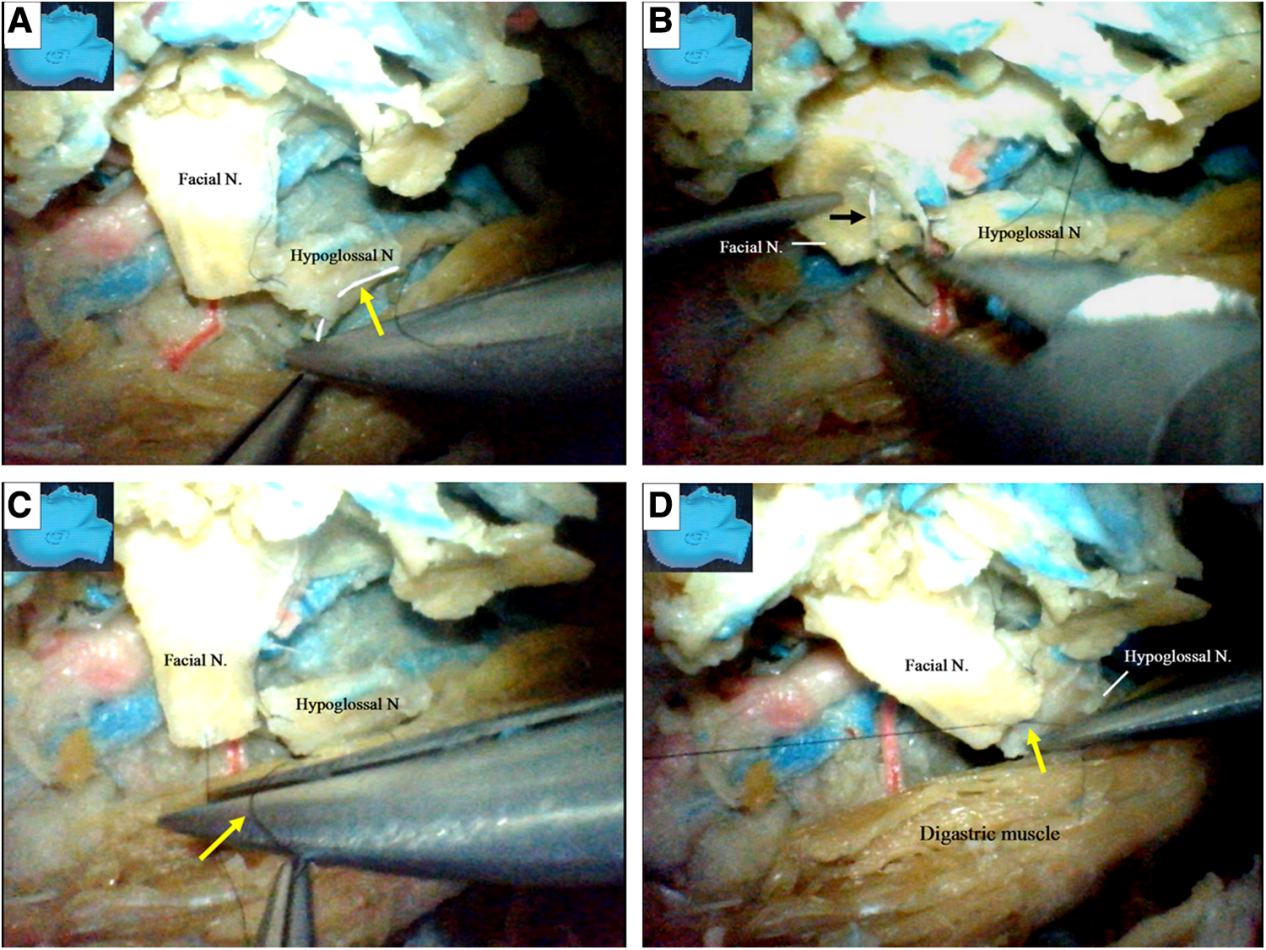
Endoscopic view: an endoscopic end-to-end hypoglossal-facial anastomosis with 11/0 nylon sutures. (**A**) The needle (arrow) is passing the hypoglossal nerve; (**B**) the needle (arrow) is then passing out of the facial nerve; (**C**) a loop is made around the needle (arrow) holder to tie a knot; (**D**) the suture is then tightened, and a throw of knot has been completed. N, nerve.

Finally, when the recycled facial nerve stumps became too short for further side-to-end hypoglossal-facial anastomosis, the hypoglossal nerves were then transected, and an endoscopic end-to-end hypoglossal-facial anastomosis simulation with 11/0 nylon sutures was performed *in situ* without using an operating microscope (Figure 11).

We found that a stump of the facial nerve, either harvested near its external genu (Figures 8B,C) or truncated immediately caudal to the facial recess (Figure 10A), could provide adequate length for a tensionless side-to-end hypoglossal-facial anastomosis without the need for further nerve dissection within the parotid gland. A facial nerve stump truncated at the stylomastoid foramen (Figure 10B), however, often could not provide sufficient nerve length for a tensionless side-to-end hypoglossal-facial anastomosis. In the 12 facial nerve stumps studied, we could only perform a side-to-end hypoglossal-facial anastomosis with some tension using 8/0 nylon sutures after an extensive dissection (Figure 10C); in the other four stumps, we failed to do so. In such cases, the hypoglossal nerve was cut, and an endoscopic end-to-end hypoglossal-facial anastomosis with 11/0 nylon sutures was performed *in situ* without using an operating microscope (Figure 10D).

Now here raises a question: could a stump be harvested near the external genu of the facial nerve replaced by a shorter one harvested immediately caudal to the facial recess? To answer this question, five aforementioned cadaver heads (10 sides) were recycled for the measurements.

**Figure 12:**
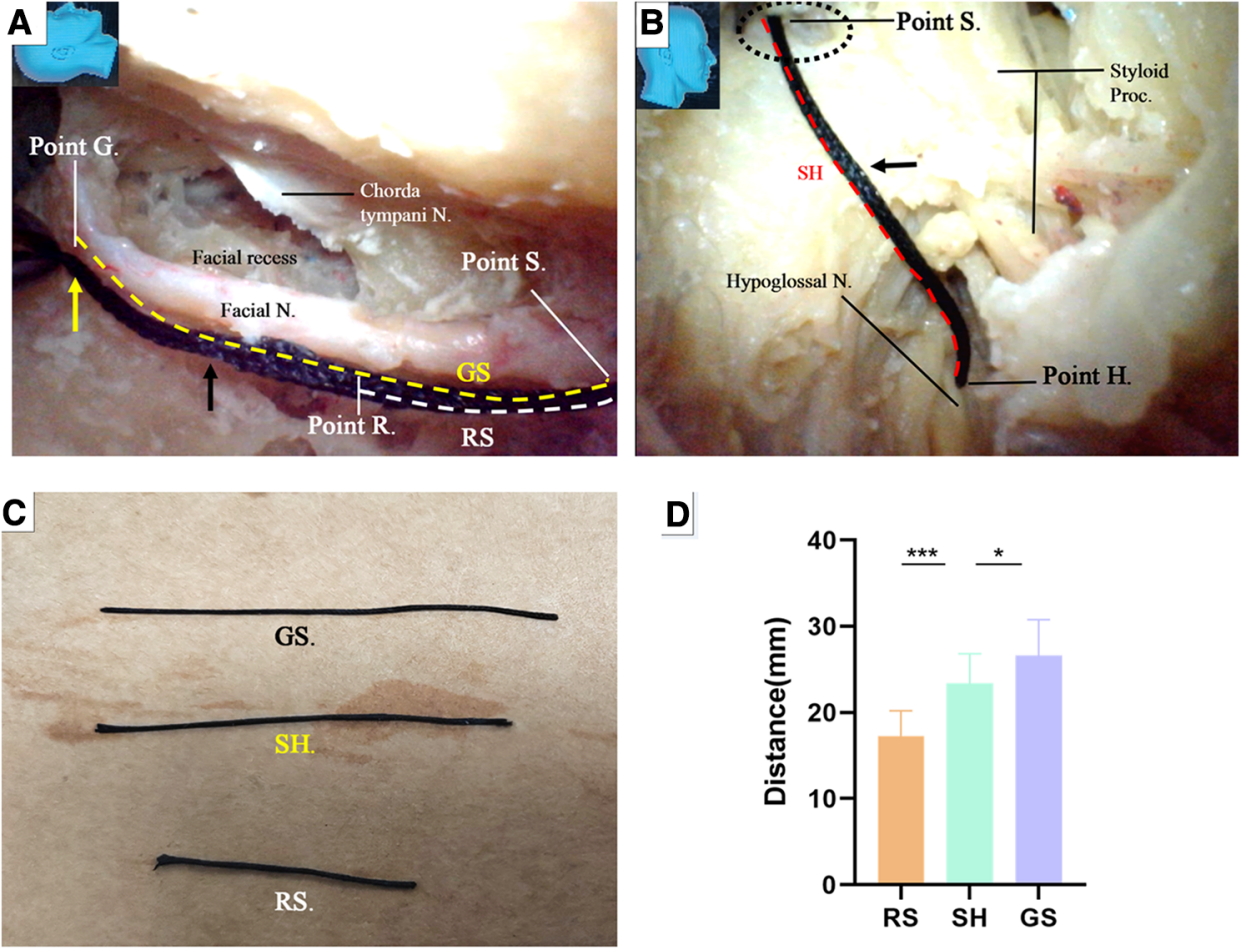
Endoscopic view: quantitative measurement. (**A**) The trajectory of the facial nerve canal was replicated with a thread (black arrow). The segment of the trajectory between the Points S and G was defined as GS (yellow dash line), representing the length of a stump harvested near the external genu of the facial nerve, whereas the segment of the trajectory between the Point S and Point R was defined as RS (white dash line), representing the length of a stump harvested immediately caudate to the facial recess, respectively; (**B**) the thread was passed out of the stylomastoid foramen (dash circle) from inside, all the way to reach the previously exposed hypoglossal nerve, replicating the trajectory between the Point S and the nerve, as short as possible. The site on the surface of the nerve touched by the tip of the thread was defined as Point H. The segment of the thread bypassing the Point S and Point H was named SH, representing the minimal length of a facial nerve stump required to ensure a side-to-end hypoglossal-facial anastomosis; (**C**) the segment of thread representing different parameters were harvested and measured with a caliper; (**D**) Chart: the data measured from both GS and RS were compared with that from SH by paired (matched) *t*-test (tails = 2), respectively. The result indicated that GE is statistically longer than the SH (*P* < 0.05) (Table); GS is statistically shorter than the SH (*P* < 0.001). N, nerve; Proc, process.

The key reference site was selected at the internal orifice of the stylomastoid foramen and defined as Point S (Figures 12A,B). The trajectory along the facial nerve canal was replicated with a thread (Figure 12A). Two harvesting sites for the facial nerve stumps were selected. One was near the external genu of the facial nerve (canal) (12 and 13) as Point G (Figures 8B, 12A), the other was immediately caudal to the facial recess as Point R (Figures 10A, 12A), respectively. The segment of the thread between the Points S and G was named GS, representing the length of a stump harvested near the external genu of the facial nerve (canal), whereas the segment of the thread between the Points S and R was named RS, representing the length of a stump harvested immediately caudal to the facial recess, respectively (Figure 12A).

Under the guidance of the endoscope, we inserted the thread through the external orifice stylomastoid foramen and reached Point S, and then placed the other end of the thread at the part on the hypoglossal nerve closest to the Point S, which is named Point H (Figure 12B). The segment of the thread bypassing the Point S and Point H was named SH, representing the minimal length of a trajectory replicable between the stylomastoid foramen and the hypoglossal nerve (Figure 12B).

The GS, RS, and SH was harvested and measured with a caliper, respectively (Figure 12C). The result was recorded. The result indicated that GS is statistically longer than the SH (*P* < 0.05) (Figure 12D), which is congruent with the aforementioned result that the length of a facial nerve stump harvested near the external genu is adequate to ensure a side-to-end hypoglossal-facial anastomosis. RS, however, is statistically shorter than the SH (*P* < 0.001), (Figure 12D), which questions the qualification of the latter stump for a tensionless anastomosis proven by a surgical simulation. The former stump is indisputably optimal. Regardless of the questioning from the results of the measurement, since the qualification of the latter stump for a tensionless anastomosis had been proven by the surgical simulation, this stump merits a substitute in case that an optimal stump is unavailable. In fact, the reach of a stump may be extended far beyond its own length via surgical manipulations to loosen and free the stump from its surrounding structures.

## Discussion

An end-to-end hypoglossal-facial anastomosis is an effective procedure for the surgical rehabilitation of facial nerve function, with postoperative improvement lasting more than 3 years; however, it is associated with disarticulation and tongue numbness, which are almost inevitable following sacrifice of the hypoglossal nerve (5). Thus, a side-to-end hypoglossal-facial anastomosis was introduced to preserve part of the function of the hypoglossal nerve (10, 11, 17).

An endoscope-facilitated surgery, with advantages including a broad surgical field, multiple surgical views, and the ability to quickly switch between surgical fields and targets, has led to breakthroughs in traditional surgical paradigms with the single-view approach, confined surgical field, and limited mobility of an operating microscope.

In this simulation study, we demonstrated that the endoscope-dominated side-to-end hypoglossal-facial anastomosis is not only feasible but may also provide several surgical advantages.

We found that with modification, our transaditus endoscopic approach (14) may offer an effective option to perform endoscope-dominated side-to-end hypoglossal-facial anastomosis surgery. The procedure began with conventional cortical mastoidectomy. Once the tympanic antrum was reached (Figure 5A), an endoscope was introduced (Figure 5B), and the surgical simulation could be successfully performed predominantly utilizing an endoscopic approach.

With the endoscopic approach, we found that dislocation of the incudostapedial joint (Figures 6G,H) and removal of the incus (Figure 6H) as performed during the traditional transaditus approach were not necessary. In fact, we preferred to use the intact incus as a landmark for the facial recess and facial nerve localization (15). Because the site of the facial nerve transection was designed distal to the incus buttress, resection of the buttress would provide no benefits to the surgery (Figure 8B).

We found that the facial nerve could often be well visualized under endoscopy without using the styloid process as a landmark, and the facial nerve stump could be harvested without fracture or retraction of the styloid process (13), as performed in traditional surgery. In rare cases, if necessary, the identification, fracture (Figures 2B,C), and retraction of the styloid process with the attached muscles could also be easily performed with the endoscopic technique.

We also found it feasible to optimize several procedures involved in classical microscopic cortical mastoidectomy under endoscopy. Under endoscopy, the superior and posterior semicircular canals did not need to be exposed, and the lateral semicircular canal was only occasionally used as a landmark. Once the ossicular chain and the lateral semicircular canal (Figures 6A–C,H) were identified, the other endoscopic surgical steps could be uneventfully performed.

Under endoscopy, the distal segment of the facial nerve at the stylomastoid foramen could be located (Figures 2A,B, 7D), and dissection involving the parotid gland could be minimized (Figure 2A). The distal segment of the facial nerve could be exposed either together with the hypoglossal nerve in the early stage of the surgery or later, when the stylomastoid foramen has been opened (Figures 7C,D). We recommend a combined intra- and extracranial management of the facial nerve.

Under endoscopy, we found that we could localize and perform surgery on the whole facial nerve much more easily due to the advantages of the endoscope, including the broad surgical field, multiple surgical views, and ability to switch between surgical fields (Figures 7C,D). The presence of the bony shelf beneath the facial nerve (Figures 6I,J) may serve as a natural barrier between the jugular bulb and the facial nerve. This barrier (Figure 6J) can be better appreciated with an endoscope, therefore helping to minimize the risk of inadvertent surgical injury to the jugular bulb.

We found that the stump of a facial nerve, harvested near its external genu (Figures 8B,C) or truncated immediately caudal to the facial recess (Figure 10A), can always provide sufficient length for a tensionless side-to-end hypoglossal-facial anastomosis without further nerve dissection within the parotid gland. In contrast, the stump of a facial nerve harvested at the stylomastoid foramen (Figure 10B) often cannot provide sufficient length for a tensionless side-to-end hypoglossal-facial anastomosis. In such situations, an endoscopic end-to-end hypoglossal-facial anastomosis is an option. The qualification of a facial nerve stump harvested both near the external genu for the side-to-end hypoglossal-facial anastomosis is supported unanimously by the results from the surgical simulation and the quantitative measurement. The qualification of a stump harvested immediately caudal to the facial recess, however, is questioned by the results of the quantitative measurement because the stump failed to reach the sufficient length to ensure the anastomosis. The explanation for the discrepancy is that the reach of a stump may be extended far beyond its length measured *in situ* via surgical dissections to loosen and free the stump from its surrounding tissues. The former stump is no doubt the optimal. Since the latter stump had been proved by the experiences of surgical simulation, this stump merits a substitute in case that an optimal stump is unavailable.

Cusimano and Sekhar (9) published a surgical strategy involving a partial hypoglossal-facial nerve anastomosis. The facial nerve is sectioned either at the stylomastoid foramen or in its mastoidal segment, the split hypoglossal nerve is dissected longitudinally over a distance of 2–3 mm, and the cut nerve ends are anastomosed. They also reported that the facial nerve could be connected to a short split hypoglossal nerve without performing a partial mastoidectomy. The feasibility of such a strategy, however, was challenged by Sawamura et al. (13). First, the hypoglossal nerve is not polyfascicular and therefore cannot be split into different fascicles, and a long split along its distal portion may result in hemiatrophy of the tongue (13, 18). Second, the split hypoglossal nerve will need to be up to 20 mm in length to be directly connected to an extra cranial facial nerve end without tension, even if the facial nerve is sectioned near the stylomastoid foramen. This challenge seems to be supported by our observations in this study (Figure 10C) (13, 18). Our results (Figure 10) support that exposure of the descending portion of the facial nerve in the mastoid cavity is necessary to achieve a direct hypoglossal-facial nerve anastomosis without using a graft. A facial nerve stump harvested immediately caudal to the facial recess (Figure 10A) can be used for a tensionless side-to-end hypoglossal-facial nerve anastomosis without further dissection in the parotid gland, whereas a facial nerve stump truncated at the stylomastoid foramen (Figure 10B) cannot. Even for a side-to-end hypoglossal-facial anastomosis with tension, extensive dissection in the parotid gland is necessary (Figure 10C), with a facial nerve stump truncated at the stylomastoid foramen, which may result in damage to the parotid gland and the distal branches of the facial nerve. The use of a facial nerve stump harvested at the stylomastoid foramen should not be recommended. In clinical practice, individualized evaluation to ensure adequate but avoid redundant length of each facial nerve stump with a nerve stimulator cannot be overemphasized.

We found that an endoscopic end-to-end hypoglossal-facial anastomosis with 11/0 nylon sutures can be performed using basic microsurgical techniques, including nerve trimming, needle passing (Figures 11A,B), and knot tying (Figures 11C,D). However, more time is required for this than the traditional microsurgical anastomosis.

With the high mobility and agility of an endoscope, several aforementioned surgical steps can be overlapped or interweaved during the procedure (Figures 6G,H, 7C,D). Most of the surgical maneuvers in this study, except for the microsurgical anastomosis, were completed with an endoscope.

## Conclusions

From this simulation study, we conclude that the transaditus approach with some modifications could potentially serve as an effective clinical option for performing an endoscope-dominated side-to-end hypoglossal-facial anastomosis, with several potential surgical advantages compared with the traditional nonendoscopic approach, and may lead to further improvements in clinical facial nerve repair surgery. This endoscope-dominated side-to-end hypoglossal-facial nerve anastomosis technique merits future clinical studies and refinement.

## Data Availability

The raw data supporting the conclusions of this article will be made available by the authors, without undue reservation.
